# From Canonical to Emancipatory Action Research: Using PrOH Modelling to Enhance Reflexivity

**DOI:** 10.1007/s11213-025-09716-3

**Published:** 2025-04-08

**Authors:** Krishna Chaitanya Balthu, Ben Clegg

**Affiliations:** Aston University, Birmingham, UK

**Keywords:** Action research, Systems thinking, Change management, Service operations, Manufacturing, Knowledge transfer

## Abstract

Action research is well recognised as an approach to transform, empower, and emancipate individuals and communities through collaborative enquiry and intervention. A central tenet of action research is to generate learning and new knowledge through the cyclical process of action and reflection. Yet, traditional action research methodologies pose limitations for thoroughly extracting learning from action due to lack of well-developed frameworks for understanding the researcher’s role and their evolving identity throughout the research process. This limitation undermines the depth of engagement with the problem context and the potential for a researcher to reflect and generate learning in real-time. Based on multiple in-depth longitudinal case studies conducted over a decade, this paper argues for the emancipation of action researchers through a new *Situated Emancipatory Action Research* (SEAR) framework developed using a novel soft systems methodology called the Process Oriented Holonic (PrOH) Modelling Methodology. The SEAR framework seeks to overcome the limitations inherent in action research by emphasising the importance of a cognitive journey for the researcher, moving from a primarily detached observer to an immersed agent of change, while continuously *reflecting-in-action*. This study demonstrates how the SEAR framework enables emancipation of both the *researcher* and the *researched* through an intertwining and mutually complementary process of *deepening* and *widening* understanding through successive action research cycles. The new SEAR framework facilitates action researchers to become emancipated from their precepts, biases and identity, towards better engaging with problem situations and extraction of new knowledge. This paper recommends further investigation and experimentation using the SEAR framework to refine and improve its application in wider action research settings.

## Clinical Trial Number

Not applicable.

## Introduction: the Limitations of Canonical Action Research

Action research is a widely utilised methodology that facilitates both organisational change and knowledge creation through the collaborative engagement of researchers and practitioners. It involves an iterative process where researchers work within real-world contexts, diagnose problems, and engage participants to enable learning about a situation (Checkland 1991; Bradbury and Reason 2001; Davison et al. 2012). Its cyclical nature allows researchers to continuously reflect on the action and refine interventions based on emerging insights. Although action research has proven effective in delivering organisational change, it has been criticised for limitations around knowledge creation and the role of action researchers and their reflexivity to create new knowledge.

**Fig. 1:**
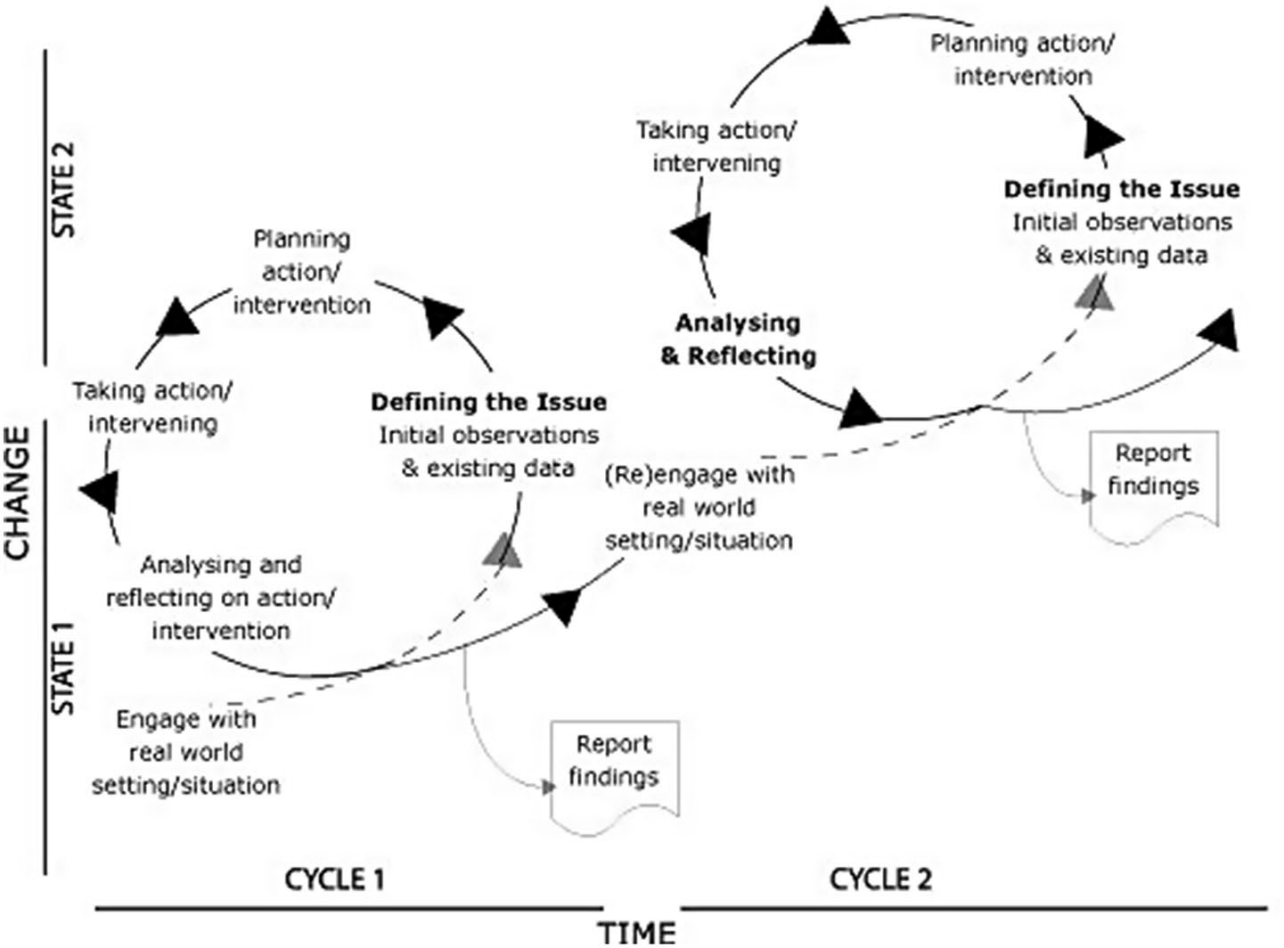
Canonical Action Research (CAR) process (Susman and Evered 1978)

A key framework within action research literature is Canonical Action Research (CAR), introduced by Susman and Evered (1978). CAR offers a cyclical model comprising of five stages as shown in Fig. 1 providing a structured process for researchers to work through organisational challenges. In this paper, CAR has been selected as the central methodology to critique, serving as a representative framework for broader action research methodologies. CAR’s structured approach, defined by successive research cycles provides a comprehensive lens through which to examine the iterative process of action research, facilitating the analysis of its strengths and limitations.

Methodologically, CAR employs an experiential, emergent, and dialogic process of knowledge creation. It iterates between action and reflection, coordinating inquiry with implementation in a fluid manner responsive to complex and dynamic contexts (Bradbury 2015). While CAR methodology has been widely applied, it has been criticised for not adequately addressing the dynamic role of the researcher within the CAR process. Literature highlights the challenges in operationalising the cyclical model and the role of theory in its enactment (Davison et al. 2012). There is little research on action researchers’ perspectives and cognitive journeys when using the CAR model at the process-level. For instance, Davison et al. (2004) indicates that CAR often positions the researcher as an external observer, which limits their engagement with the organisational context, and hinders their ability to capture the complexities of organisational behaviours. Also, by focusing on the organisation as an object of study, a researcher’s subjectivity and embodied experiences are frequently overlooked, reducing their capacity for meaningful reflection.

Researchers engaged in CAR often encounter the recurring challenge of extracting learning from action, which is commonly referred to as ‘reflection’. They also need to distinguish between two types of reflection: ‘reflection-on-action,’ defined retrospectively, and Schon’s (1983) ‘reflection-in-action’. Understanding reflection-in-action can be complex, and scholars have noted that this can sometimes be conflated with reflection-on-action, suggesting that further exploration is required. For instance, Yanow and Tsoukas (2009) raise pertinent questions, such as, “How can reflection-in-action theorizing overcome its cognitivist bias?” and “What does this process entail?” These questions lie at the heart of action research challenges which this paper seeks to address. The limitations of CAR in effectively extracting learning from action, particularly in the context of reflection-in-action, have been a subject of scholarly debate. For instance, CAR’s emphasis on detached observation can create a temporal gap between observed action and reflection which may compromise the accuracy and authenticity of reflection, as the immediacy and intensity of the experience diminishes over time. Coughlan and Coghlan (2002) highlight the importance of conscious and deliberate enactment of action research cycles and contend that the temporal separation may impede researchers’ capacities to reflect in the heat of the moment, affecting the depth and accuracy of insights gained and reported. This concern about temporal separation and its potential impact on researhers’ abilities to reflect and gain insights in real-time can be understood through the lens of *situatedness*, as articulated by Haraway (1988). This notion of situatedness emphasises that knowledge is inherently embodied, partial, and situated within specific contexts and perspectives. In the context of action research, this means that knowledge generated is shaped by unique perspectives, experiences, and contexts of researchers and participants, which is often referred to as the ‘*weltanschauung’* (the ‘world view’) in systems thinking literature. Such temporal separation between the action and reflection phases of the action research cycle may create a disconnect between researchers’ embodied experience in the moment and their subsequent reflection and interpretation.

Limitations in capturing the ‘situatedness’ of researchers’ embedded in the problem situation are noteworthy and are a concern highlighted by Bradbury and Reason (2001). CAR, as critiqued by previous researchers, is limited in adequately recognising the dynamic and context-dependent nature of reflective practice (Bradbury and Reason 2001; Davison et al. 2012) as the complexity and ever-evolving nature of real-world situations which demands an approach to reflection that is not only adaptive but deeply situated within the specific context at hand. Researchers argue for a more subtle understanding of action research that emphasises the need to move beyond linear problem-solving models and embrace the intricacies of situatedness and reflection-in-action (Greenwood and Levin 1998; Bradbury and Reason 2001; Kemmis 2008; McNiff 2013) Also, as CAR is criticised for its tendency to adopt linear approaches and for overlooking plurality in how individuals think and know Heron and Reason (2008) argue for embracing a broader epistemological perspective that accommodates various ways of understanding and engaging with the complexities of reflective practice.

Rooted in the above critique, this paper seeks to advance the discourse on action research methodologies. It delves into the development and application of a recently published ‘situated-reflective-agent’ model (Balthu and Clegg 2021) resulting in its extension to more comprehensive Situated Emancipatory Action Research (SEAR) demonstrating that action research is *emancipatory* not only for the researched organisation but also for the researchers themselves. Emancipation, as used in this paper, draws on definitions offered by various scholars including Carr and Kemmis’ (1986) who emphasise action research’s transformative potential of going beyond problem-solving to empower individuals and groups by enabling them to critically examine and challenge their own practices, assumptions, precepts, and biases, including researchers and the researched. Emancipation has been further explored by McTaggart (1994), who highlights the importance of participatory action research in fostering critical reflection and transformation, McCutcheon and Jung (1990) who discuss how action research provides the opportunity for individuals and groups to engage in dialogue and reflection leading to empowerment and self-determination; and, Grundy (1988) who emphasise that at core of emancipatory action research lies the spirit to enable participants to question and alter underlying structures that constrain them.

This paper brings forth findings from dozens of in-depth longitudinal case studies, conducted by the authors in various organisations over a decade aiming to demonstrate the practical application of the new framework, offering valuable insights about its potential to bridge existing gaps in traditional action research methodologies. Two of these case studies will be used in this paper to explicate the new SEAR framework.

## Literature Precis: Action Research and its Limitations

Carr and Kemmis (1986) identified at least three distinct types of action research, each characterised by distinct aims and relationships between researchers and practitioners. Table 1 illustrates these three types with their respective features.

**Table 1:** Types of action research

Type of Research	Aims	Facilitators Role	Researcher – Participant Relationship
1. Technical	Informing	Expert	Participants dependent on researcher
2. Practical (interpretive)	Understanding	Participation	Process-oriented consultation
3. Emancipatory	Transformation	Moderator	Collaboration to expose injustices

Source: Carr and Kemmis (1986*)*

The distinction among these three types of action research lies not in their methodologies but in the underlying assumptions and worldviews of participants, leading to variations in how methodologies are applied. Within the action research process, researchers actively engage in action, assuming the role of insiders who influence group actions. Therefore, pragmatism serves as a philosophical foundation of action research (Greenwood and Levin 1998), with terms such as “intervention”, “collaboration”, and “interactivity” commonly used to characterise it (Eikeland 2012, p.10). However, action research is also recognised for its dual objectives: (i) generating knowledge and action directly applicable to specific groups and (ii) empowering individuals at a deeper level and emancipating them by facilitating the construction and utilisation of their own knowledge (Reason 2001; Grundy 1988). So, besides pragmatism, action research also requires reflection whatever type of action research one uses.

### Critique of Canonical Action Research

Canonical Action Research (CAR) can foster organisational and societal change but has been criticised for not fully capturing the complexities of reflective practice (Coghlan and Brannick 2014). While action research serves a broader purpose, researchers practicing it encounter inherent dilemmas. One such dilemma is the dual objective of addressing a research question while simultaneously meeting a practical need (Rapoport 1970). Additionally, action researchers often face criticism for their failure to disclose and deliberate upon intellectual frameworks that may inform their projects Checkland (1981, p.400). Checkland (1995, p.2) strongly “advocates the need for an intellectual framework, declared in advance, within which learning is defined. Without such a framework, action research risks becoming indistinguishable from mere action and anecdotal accounts of what happened”. Checkland (1985) delves further into the above process by proposing the FMA model which posits that an Action Research intervention must be guided by a purposeful strategy or methodology (M) supported by a set of related concepts within a theoretical framework (F), to enhance a particular situation or area of application (A).

Other researchers emphasised the need to re-evaluate power dynamics within the research process. For instance, Carr and Kemmis (1986) argue that CAR often neglects the unequal power relations between researchers and participants, potentially leading to the imposition of researcher perspectives on the researched. This power differential can hinder the co-creation of knowledge and the genuine engagement of participants in the reflective process. Therefore, there is a growing call for action research methodologies to actively address and redress these power imbalances, promoting a more egalitarian and participatory research approach (Carr and Kemmis 1986; Davison et al. 2004).

The temporal dimension of CAR has also been criticised by Coghlan and Brannick (2014) as they contend that the cyclical nature of canonical action research might not adequately capture the dynamic nature of contemporary organisational challenges. Extending the durations of action research cycles may lead to a lag in responding to rapidly changing situations, potentially rendering research outcomes less relevant. This critique suggests the need for more agile and adaptive action research methodologies that align with the accelerated rate of change in today’s organisational environments.

### Reflection-in-Action

Argyris and Schön (1974, p.221) describe action research as ‘action science’ and emphasize that ‘consciousness in the midst of action’ by a researcher is a fundamental principle of action inquiry. One prominent limitation of CAR is its implicit cognitive emphasis, which aligns with Schön’s (1983) critique of reflection. Schön argues for a broader understanding of reflection, introducing the concept of “reflection-in-action”, which emphasises real-time reflection during the action itself, rather than reflecting only after the fact. This perspective recognises that practitioners embedded in the problem situation often engage in problem-solving reflexively in real-time situations, incorporating tacit knowledge and skills. An action researcher’s inclination towards detached observation neglects this subtle, embodied, and context-dependent form of reflection (Griffin et al. 1999). Also, as noted by Susman and Evered (1978), there is a historical predisposition in action research towards a cognitive focus, emphasising rational problem-solving processes over the more intricate and subtle aspects of human action. This bias tends to overlook the intricate interplay of emotions, perceptions, and contextual factors that shape decision-making and actions in real-time situations. Such oversights hinder the depth and richness of reflection-in-action, limiting a researcher’s ability to grasp tacit knowledge and subtleties embedded in the ongoing process. The work of Reason (1994) further accentuates the cognitivist leanings of CAR, emphasising the need for a more holistic and embodied approach to reflection. Carr and Kemmis (1986) emphasise the importance of researchers’ reflexivity, highlighting the need to critically examine their own assumptions and biases. Bradbury and Reason (2001) extend this discourse by advocating for a more participatory and collaborative role for the action researcher, challenging the conventional notion of the researcher as a detached observer arguing for a more engaged and reflexive stance. In this context Checkland and Holwell (1998) further emphasise the importance of ‘*recoverability’* which means making the research process explicit and traceable, allowing an outside observer to follow the reasoning and decisions of a researcher. This ensures transparency and accountability in dynamic research environments, where researchers’ ongoing engagement with the situation shapes their evolving understanding. Similarly, the idea of ‘*first-person inquiry*’ (Torbert 1998; Reason & Bradbury 2001; Taylor 2004) also referred to as ‘*first-person action research*’ (Marshall 2004), highlights the essential role of researchers’ direct involvement and reflection in the research process, calling for a deeper understanding of their own biases and influence on the research. According to Torbert (1998) modern science has tended to favour ‘third-person’ (objective) research over ‘first-person’ (subjective) and ‘second-person’ (intersubjective) research. These critiques emphasise an evolving understanding of action researchers’ role, from an observer to an active agency of ‘first-person’ change, requiring a novel and innovative approach to address the complexities of real-world problem-solving.

### Evolving Identity of the Action Researcher

Recent studies discuss how researcher identity is shaped through continuous engagement with the research context. Castelló et al. (2020) provide a comprehensive review of two decades of research on researcher identity, identifying key dimensions such as the transitioning among identities, the balance between continuity and change, and the development of personal identity over time. These insights posit that an action researcher’s identity is not static but evolves through iterative cycles of reflection and engagement. This dynamic transformation is essential in understanding how researchers become more immersed in a research setting, shifting from detached observers to active agents of change. Hakkarainen et al. (2023) similarly highlight the fluidity of researchers’ professional identities and how they shift when they become more engaged with the research context. Cunliffe (2003) argues that researcher identity is continually constructed through interactions with participants and the research context, highlighting the evolving nature of the researcher’s role. As Stowell (2024) notes, our understanding of a situation is continually updated through reflection on our actions, but ultimately, it remains rooted in our personal experience of the world, which is always in flux. This aligns with Checkland and Poulter’s (2006, p. XV) notion of ‘multiple interacting perceptions of reality,’ underlining the dynamic and shifting nature of the problem context as well as researchers’ evolving understanding within the action research process.

This study shows that researchers find their professional identity challenged as they engage deeply with the research topic, shifting from conventional roles into those that align more closely with the research setting itself. Similarly, Weiner-Levy and Abu-Rabia-Queder (2012) discuss how positionality of being an insider or outsider plays a crucial role in a researcher’s identity formation, emphasising that these positions are not fixed but change throughout the action research process as a researcher’s relationship with participants deepens.

These shifts in *identity* and *positionality* are intertwined with reflexivity, a core component of action research. Alvesson and Sköldberg (2009) emphasise that reflexivity allows researchers to critically reflect on their own biases, assumptions, and values, which are shaped by their evolving identities. Reflexivity is crucial in participatory and collaborative action research, where a researcher’s evolving identity influences not only their role within the research process but also the relationship they build with research participants. Understanding the fluid nature of researcher identity, alongside the continuous process of reflection, is essential for dealing with the complexities of action research, where researchers and participants co-create knowledge through ongoing interaction. These insights highlight the need for a deeper exploration of how a researcher’s identity evolves over time and how these changes affect the research process and its outcomes. Embracing the fluidity of identity and positionality, and inculcating reflexive practices, will be key to improving the effectiveness and impact of action research methodologies.

As substantiated by the above discussion, action research still has several limitations to fully realise its transformative and emancipatory potential. These limitations emphasise the need for innovative methodologies to overcome the above-mentioned challenges on researchers’ identity, namely *positionality* and *reflexivity*, and offer a more comprehensive and adaptable framework for organisational inquiry and change. A framework for addressing some of these limitations is discussed below.

### Development of the Situated-Reflective-Agent Model

To further illustrate the role of a researcher in action research, the authors have previously coined the term ‘Situated-Reflective-Agent’ (SRA) as a proxy to ‘action researcher’, to try to address the issue of cognitivist bias as well as to enhance reflection-in-action (Balthu and Clegg 2021). The concept of ‘situatedness’ refers to an agent embedded in the environment, emphasising the importance of direct engagement with the research context. This means that a researcher, immersed in the ‘problem situation,’ reflects upon their experiences and actions within the context, allowing their understanding to evolve. In doing so, a researcher’s identity is shaped by their engagement with the research process and the participants, leading to a more dynamic, adaptive role that goes beyond simple observation or detachment.

**Fig. 2:**
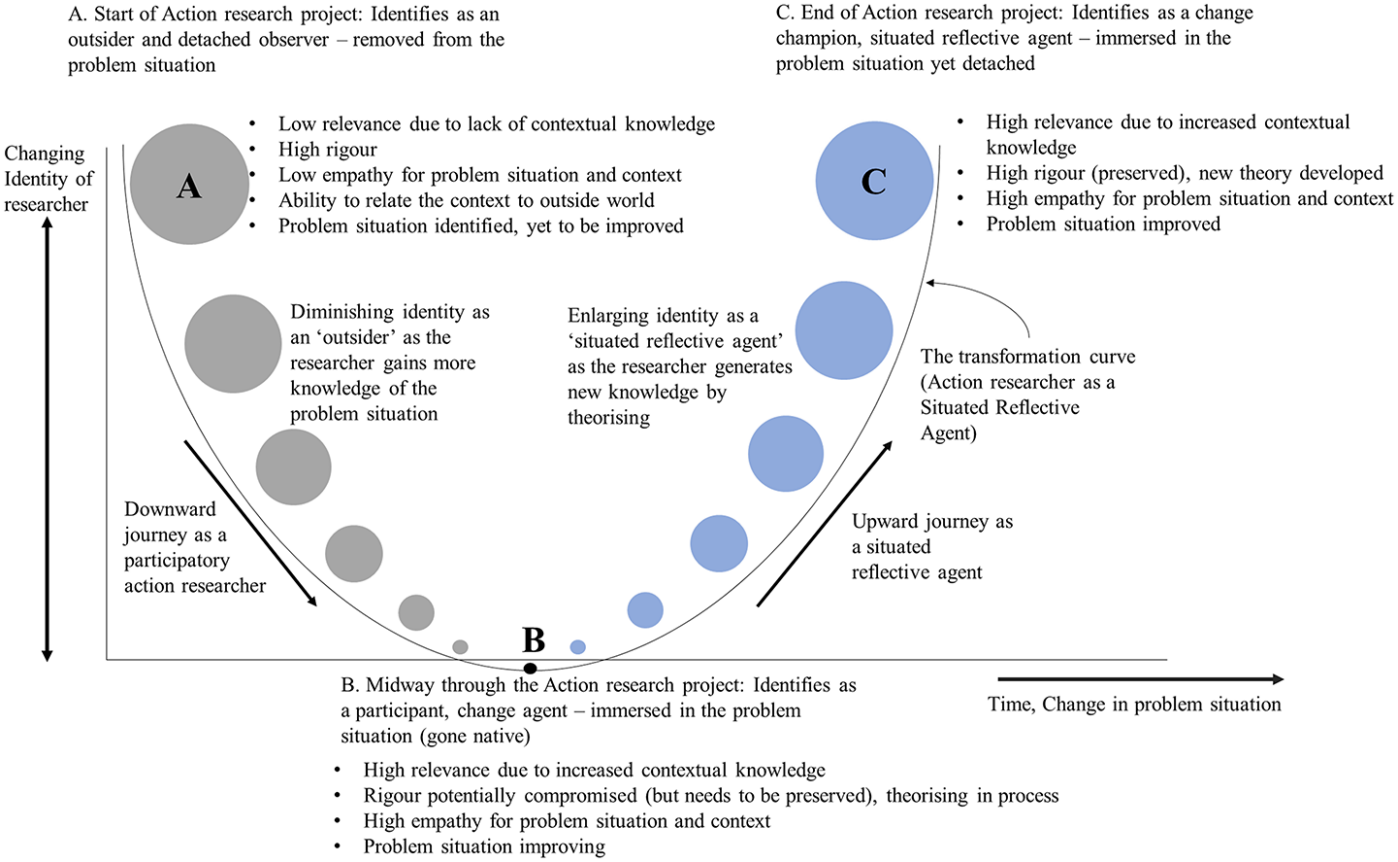
Evolving Identity of the Action Researcher into a ‘Situated-Reflective-Agent’. Source: Balthu and Clegg (2021

The philosophical basis for the Situated-Reflective-Agent (SRA) model is discussed along with a detailed explanation of the cognitive journey in a recent paper by Balthu and Clegg (2021). The SRA model is presented in Fig. 2 to provide the background for the extended framework presented later in this paper.

Previous research proposes that the essence of the ‘Situated-Reflective-Agency’ (SRA) model was to initially position a researcher in the place of detached observer (at Point A) which is akin to the *pure inquiry* of Schein (1999). As a researcher becomes more immersed and reflective in the process of inquiry, progressing to Point B, where the transition occurs from being an outsider to “going native” with the organisation, gaining deeper engagement and understanding as demonstrated in the downward journey. This downward journey represents Polanyi’s (1966) concept of *tacit knowledge* as a researcher is developing in-situ. The researcher then gradually emerges at Point C through an upward journey as a Situated-Reflective-Agent, fully integrated within the organisation through praxis and continuously reflecting-in-action.

The SRA model provides a valuable stepping stone for conducting rigorous action research by emphasising the importance of embedding the researcher within the organisational context and adopting an intentional reflective practice. However, the SRA model stops short of providing concrete guidance on translating its principles into a comprehensive research design that can support impactful organisational transformation and therefore a missed opportunity to address the limitations of action research approaches discussed above. Thus, the remainder of this paper explains how an extended framework has been developed to tackle the constraints of CAR.

## Methodology: A Novel Approach to Enhance Canonical Action Research

This study uses two in-depth longitudinal case studies underpinned by CAR, one from the service sector and one from the manufacturing sector, intending to capture a broad spectrum of organisational processes and challenges.

Each case study was a two-year engagement with an organisation, allowing for a deep exploration of their respective contexts, challenges, and opportunities. The qualitative methods employed included interviews with key stakeholders (*n* = 35), focus groups comprising staff from cross-functional groups (x9), and PrOH Modelling workshops (x10), in total across both the organisations. These methods were chosen to gather rich and context-specific data, facilitating a comprehensive understanding of the dynamics within each organisation. A qualitative research approach, as advocated by Denzin and Lincoln (2005), provided an in-depth understanding of complex phenomena by capturing the nuances of human experiences and organisational dynamics. The interviews and focus groups facilitate the exploration of participants’ perspectives, allowing for a deeper comprehension of their attitudes, motivations, and challenges.

### Case Study Organisations

The first case study organisation operates in the legal services sector, with a history of over 140 years as a full-service law firm, employing around 250 staff across eight specialised departments. This law firm faces various challenges, including the imperative to enhance operational efficiency, implement innovative pricing and service delivery practices, devise novel people management strategies, and foster a cultural shift towards greater inclusivity across roles and departments.

In contrast, the second case study organisation is a small-to-medium (SME) sized manufacturing company, specialising in designing and manufacturing customised retail displays for renowned global brands and retailers. With a staff of 50 and a multinational clientele, this company encounters specific challenges such as capacity constraints, the impact of legacy process management on service delivery and profitability, and challenges related to workflow and scheduling. The complexity of the manufacturing processes and the demand for bespoke retail displays created layers of organisational challenges.

These two case study organisations, law firm and the manufacturing company, represent contrasting sectors with unique operational environments. The examination of these organisations, guided by the PrOH Modelling Methodology, provides insights into how limitations of action research could be remedied towards delivering more impactful and emancipatory change. The longitudinal aspect of the case studies is crucial for capturing changes over time, offering insights into the evolving nature of both organisational processes and researcher identity. Longitudinal studies are particularly valuable in tracking change and development, allowing the researcher to observe how organisational challenges and responses unfold over extended periods (Yin 2017).

The original SRA model was formulated on the initial law firm study which has then been extended into the new (SEAR) framework using the second case study from the manufacturing company. By focusing only on the manufacturing company this research has explored the nuances of the SRA model (previously grounded in a service organisation) within varied contexts, particularly within the manufacturing sector. By focusing on this specific case study, the research offered a more in-depth analysis of the challenges faced by SMEs in the manufacturing domain, such as capacity constraints, legacy process management, and workflow complexities. This focused approach allowed for a more thorough exploration of how the SRA model can be adapted and extended to not only address these unique organisational challenges effectively, but to also help in developing a more comprehensive approach to move away from the limitations of traditional action research. It is important to note that while insights from the first case study organisation, the law firm, were instrumental in developing the SRA model initially, this paper will primarily discuss findings from the manufacturing company to illustrate the refinement and applicability of the SRA model in diverse operational environments.

### Process Oriented Holonic (PrOH) Modelling

The basis of this paper’s methodological approach was the use of Process Oriented Holonic (PrOH) Modelling Methodology, a novel methodology derived from Soft Systems Methodology by Clegg (2007). The PrOH Modelling Methodology has been developed as a transformative methodology rooted in the principles of Soft Systems Methodology (SSM) and tailored for business process redesign (Clegg 2007; Clegg and Shaw 2008). The criticism of conventional process mapping techniques is that process maps are overly reductionist, especially when modelling intricate conceptual models of change such as Human Activity Systems (HAS) (Checkland 1981), and this criticism is one of the main motivations for why the novel PrOH Modelling Methodology was developed. Reductionism is a way of simplifying complex phenomena by breaking them down into smaller, more understandable parts. Reductionist approaches, are often referred to as ‘hard systems’ approaches (Checkland 1981; Wilson 2001) and often overlook the interactions and emergent properties of systems, potentially leading to an incomplete understanding of their behaviour and dynamics, and ‘hard’ systems approaches are criticised by proponents of ‘soft’ systems approaches such as Churchman (1979), Checkland (1981), and Ackoff (2006) who favour more holistic understanding engulfing multiple perspective of human factors instead of overly simplified reductionist relationships.

Checkland (1999, pp.A7-A9) argues that applying “hard” systems without considering diverse perspectives could lead to inappropriate solutions for the actual problem. SSM, grounded in interpretive philosophy, assumes that individuals or groups interpret their situations rather than adopting the detached, objective view of a priori systems typical of a view endorsed by “hard” systems. This is a key premise of PrOH Modelling, which builds on SSM’s principles of building purposeful conceptual activity models to understand its systemic success factors (SSFs). SSM is designed to facilitate learning about the problem situation, where participants engage in purposeful action that is meaningful to them, whether goal-seeking or otherwise. Specifically, in PrOH Modelling the purpose is to understand how to change an organisational system for the better.

**Fig. 3:**
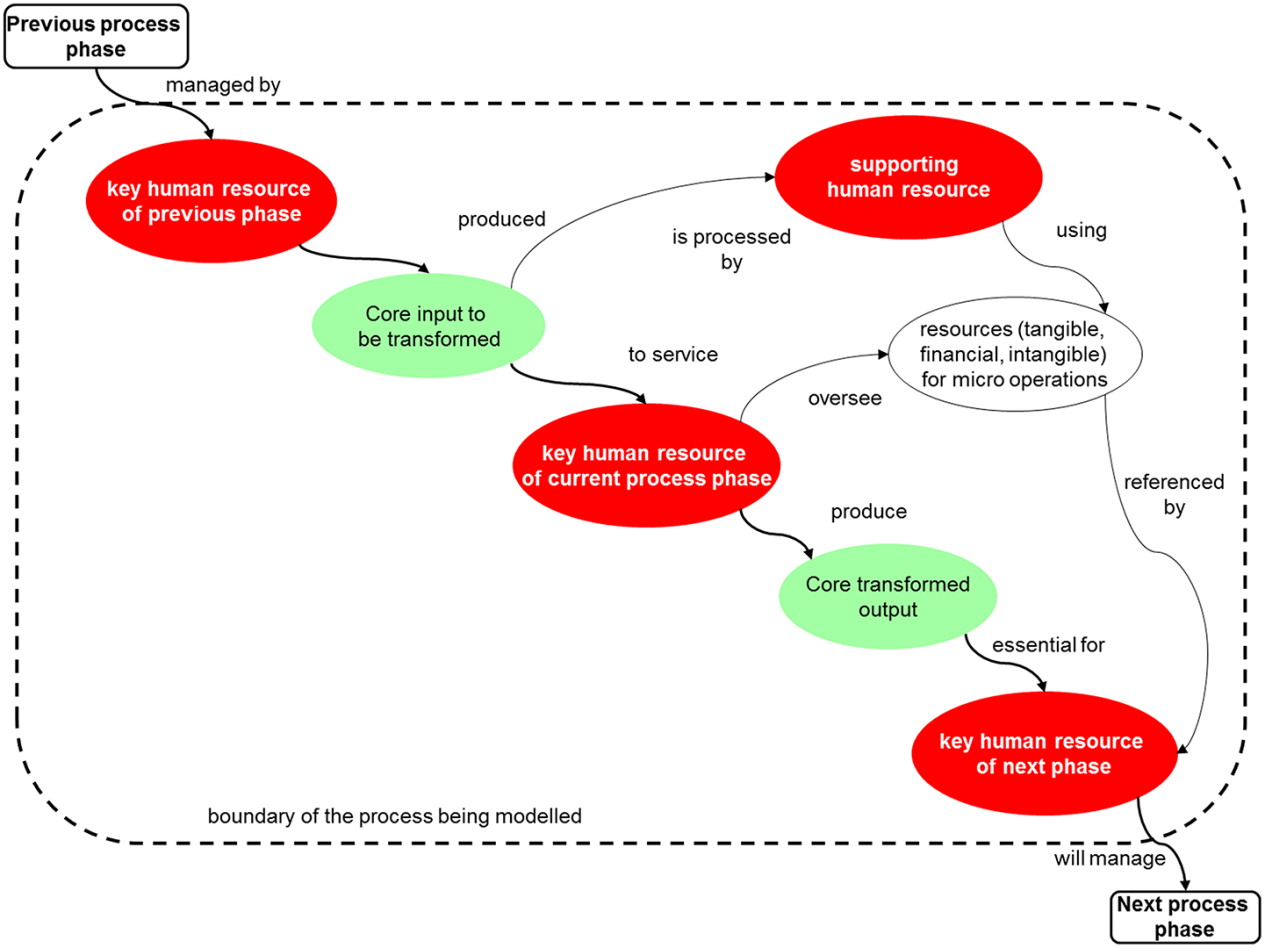
The PrOH Holon Template – “key human resource of current process phase produce core transformed output”. Source: Clegg and Shaw (2008)

The PrOH Modelling Methodology addresses reductionism, and its limitations as previously discussed, by providing a *holonic* lens that elicits systemic success factors using natural language and minimal codification by using a *holonic* template, as shown in Fig. 3. A *holon* is an entity that functions both as a whole system and as part of a larger system, addressing the conundrum of what constitutes a part and what constitutes a whole in a system. The term “holon” was introduced by Arthur Koestler in *The Ghost in the Machine* (1967), where he describes holons as “Janus-faced entities,” referring to their dual role (e.g. simultaneously being a ‘part’ and a ‘whole’). Koestler coined the term from the Greek *holos* (whole) combined with the suffix *-on*, suggesting a part or particle, as in prot*on* or neutr*on* (Koestler, 1967, p.48). This distinctive feature of PrOH Models is particularly valuable in the context of action research where the complex interplay of perceived conceptual human activity systems of change for organisational processes demands a holistic (holonic-based) representation (Coghlan and Shani 2014).

Rigorous action researcher engagement facilitated by PrOH Modelling, during the action research process, serves as a robust intervention mechanism. Unlike traditional process mapping, PrOH Modelling, as Sampson (2012) highlights, becomes imperative in conceptualising, visualising, and analysing service operations characterised by high human labour intensity and customer contact. The inherent ability of PrOH Modelling to bring out hidden and emergent properties of systems aligns with the participatory nature of action research. The PrOH Model ‘holon,’ template is akin to Checkland’s SSM ‘root definition,’ (Checkland and Scholes 1990), as shown in Fig. 3. It defines essential systems (or more precisely ‘holonic’) components, encompassing inputs/outputs (green bubbles), data, intangible elements, systemic success factors (white bubbles), human actors (red bubbles), and their interactions within a process (a system boundary – shown by the large dotted line box). These components form the minimum viable system necessary for understanding change. This comprehensive representation ensures that a PrOH Model not only captures observable phenomena but also delves into the underlying dynamics, facilitating the extraction of both practical insights for immediate change and theoretical insights for the development of an adaptable model of intervention. It is important to stress that a PrOH Model is not intended to be a model of an actual real-world process ‘as-is’ or ‘to-be’ but a model of the systemic success factors of its change from ‘as-is’ to ‘to be’ and so is *ipso facto* a soft systems model of a *conceptual system of change*.

The application of the PrOH Modelling Methodology has resulted in sets of PrOH Models known as a ‘holarchies’. PrOH Models have served as instruments to systematically extract and articulate the intricate interactions among individuals, systems, and both tangible and intangible entities within the realms of legal services and manufacturing operations, spanning various departmental and organisational levels. Drawing upon the *abstraction* and *enrichment* principles inherent in the PrOH Modelling Methodology (Clegg 2007), purposeful sets of PrOH Models have been developed. These models brought to light *systemic success factors* (SSFs) crucial for instigating change within both legal services and manufacturing firms. There is previous evidence of applying the PrOH Modelling Methodology to successfully elicit SSFs in a variety of business environments (Clegg et al. 2020; Clegg 2020).

PrOH Models are used in ‘storyboarding’ workshops, strategically designed to secure buy-in for improving the identified systemic success factors for change where stakeholders are involved in crucial decision-making about the system being changed, and where a PrOH Modeller is in a facilitator role rather than in an omnipotent autocratic role. This intervention-based approach aligns with the principles of action research put forth by Davison et al. (2004) ensuring stakeholders are actively engaged in the process of conceptualising and visualising systemic improvements (Coghlan and Shani 2014). ‘Storyboarding’ workshops have not only fostered collective understanding but also helped establish action teams. These teams, armed with a shared vision derived from the PrOH Models and the workshops, then felt empowered to collaboratively develop targeted solutions and, crucially, have strengthened agency to implement changes. A seamless transition from modelling to participatory workshops and subsequent action teams also embraces the holistic and iterative nature of the canonical action research (CAR) methodology, ensuring that theoretical insights from PrOH Modelling translate into practical and impactful changes within the organisational context.

Thus, PrOH Modelling has served as a powerful tool to navigate the intricacies of action research, offering a structured framework for visualising and analysing human activity systems within selected organisations while PrOH Models per se provide a visual representation of these organisations’ systemic success factors.

### Implementing Canonical Action Research

Within each case study organisations, each department/service line formed its own action research cycle lasting approximately 12 weeks in total; cycles were staggered and overlapping. The CAR methodology involved the five steps: (1) Defining the Issue, (2) Action Planning, (3) Action Taking, (4) Evaluating, and (5) Specifying Learning.

In the process of using CAR, the five principles laid out by Davison et al. (2004) were used as shown below, the:

1. Principle of the researcher–client Agreement
2. Principle of the cyclical process model
3. Principle of theory
4. Principle of change through action
5. Principle of learning through reflection

The application of CAR principles and stages to each case is presented in Table 2.

**Table 2:** CAR Execution model with different stages and actmodel with different stages and activities

CAR Stages	CAR Principles	Research activities based on PrOH modelling methodology to create a conceptual model of change
Defining the Issue	***Principle of the researcher-client agreement*** ensures that the researcher and client have a mutual understanding of the research goals.	Stakeholder interviews, drafting and refinement of maps, agreeing systemic success factors
Action Planning	***Principle of the cyclical process model*** involves cycles of action and reflection.	PrOH Model storyboarding workshop, action team formation
Action Taking	***Principle of change through action*** emphasises practical interventions and problem-solving.	Action teams deliver tasks, target operating model development
Analysing and Reflecting	***Principle of theory*** allows theoretical insights to guide the reflection on progress and learning.	Target operating model refinement, consolidation team meetings, evaluating systemic success factors
Reporting Findings	***Principle of learning through reflection*** serves as a critical mechanism for understanding and improving the researchers’ actions, decisions, and interventions.	Report on lessons learnt, final dissemination and handover

### Canonical Action Research Imbued with the PrOH Modelling Methodology

The PrOH Modelling Methodology played a pivotal role in supporting the overall research approach. By creating visual representations of change systems of human activity systems, PrOH Modelling enabled action researchers to elicit both tangible and intangible systemic success factors. The iterative and participatory nature of the PrOH Modelling Methodology facilitated continuous reflection-in-action, aligning with the principles of action research (Reason & Bradbury 2001). The development of PrOH Models served not only as a diagnostic tool but also as a catalyst for intervention, promoting engagement and collaboration among stakeholders.

The above approach allowed the visualisation of intricate feedback loops between individuals and processes, aligning with the participatory stance advocated by Kemmis (2008). PrOH Modelling, as proposed by Clegg (2007), offers a structured method for representing dynamic systems, making it well-suited for grasping the dynamics of changing organisational forms. For example, legal service firms classified in literature as Professional Service Firms (PSFs) are considered complex and distinct organisations (Blau and Scott 1962; Lorsch and Mathias 1987; Nordenflycht 2010). Likewise previous researchers have noted that “PSFs are organisationally fragmented” (Alvesson and Karreman 2004), and that, “Managers view [the] management of PSFs [to be] like herding wild cats”. Similarly other researchers liken managing PSFs to “making ten or twenty racing horses pull a cart together” (Lowendahl, 1997 p.63); also, that PSFs are often portrayed as a “… fashionable store – a flash brand on the outside with a lot of franchises on the inside” (Morris and Malhotra 2002 p.16). These observations on PSFs are given to bring readers’ attention to the importance of the human factors in change management that were successfully addressed in CAR imbued with PrOH Modelling.

Within a CAR and intervention-based context, the PrOH Modelling Methodology not only offered a lens into a complex organisational setting but actively contributed to reshaping organisational systems, aligning with the principles and praxis central to action research (Reason & Bradbury 2001; Eden and Huxham 1996). For example, Bradbury-Huang (2010) advocates that action research, “*proceeds from a praxis of participation*,* is guided by practitioners’ concerns for practicality [real-life problems]*,* is inclusive of stakeholders’ ways of knowing [joint-meaning construction]*, *helps to build capacity for ongoing change efforts [workable solutions]”.*

### Data Collection and Analysis

In the context of action research, the richness of the data is inherently intertwined with the participatory nature of the methodology. Coghlan and Brannick (2014) assert that data collection during action cycles is not a detached, observational process but instead constitutes an intervention in its own right. This approach aligns with the philosophy of qualitative research, emphasising the importance of contextual understanding and the co-creation of knowledge between researchers and participants (Creswell & Creswell, 2017). This deliberate sampling strategy enhances the diversity of data, covering a comprehensive spectrum of service and manufacturing operations.

Moreover, insights based on data from two distinct companies enriched the study by bringing out contextual variations and commonalities in the application of the newly proposed framework discussed in the subsequent sections. The diversity in organisational contexts ensures that the model is not only applicable in specific settings but possesses broader utility across different business environments. This aligns with the qualitative research principles of transferability and generalisability (Yin 2017). In essence, the data collection approach adopted in this study, drawing on the depth and richness of qualitative research methods, is well-suited to the intricacies of action research and the development of the new framework to comprehensively address some of the longstanding criticisms on action research.

## Implementing the PrOH Modelling Methodology: the Case of the Production Department

The production department serves as the operational heart of the organisation, responsible for translating raw materials and design specifications into finished display boards that are used in retail stores for showcasing products. This department has 11 employees, each working on various production equipment that involves cutting, printing and activities such as material handling, machine configuration and quality control. The systemic success factors for production department are on time completion of orders, quality of display boards, and safety of staff.

### Defining the Issue

Through interactions with production staff within the department, individual process flow maps were created. These maps aim to establish and illustrate the operational process flow, aiding in the identification of any potential fail points. These maps were validated and refined through in-depth interviews with key participants, each lasting 45–60 min, ensuring an accurate understanding of the workflow forming raw data from which to produce the consolidated PrOH Models containing multiple-tenable viewpoints and systemic success factors for change. The following high-level categories of issues were identified during the analysis: scheduling, communication, quality control, inventory and stock management, record keeping and logging. These categories of issues are discussed in more detail in Table 3, where the SSFs of change are outlined.

### Action Planning

**Fig. 4:**
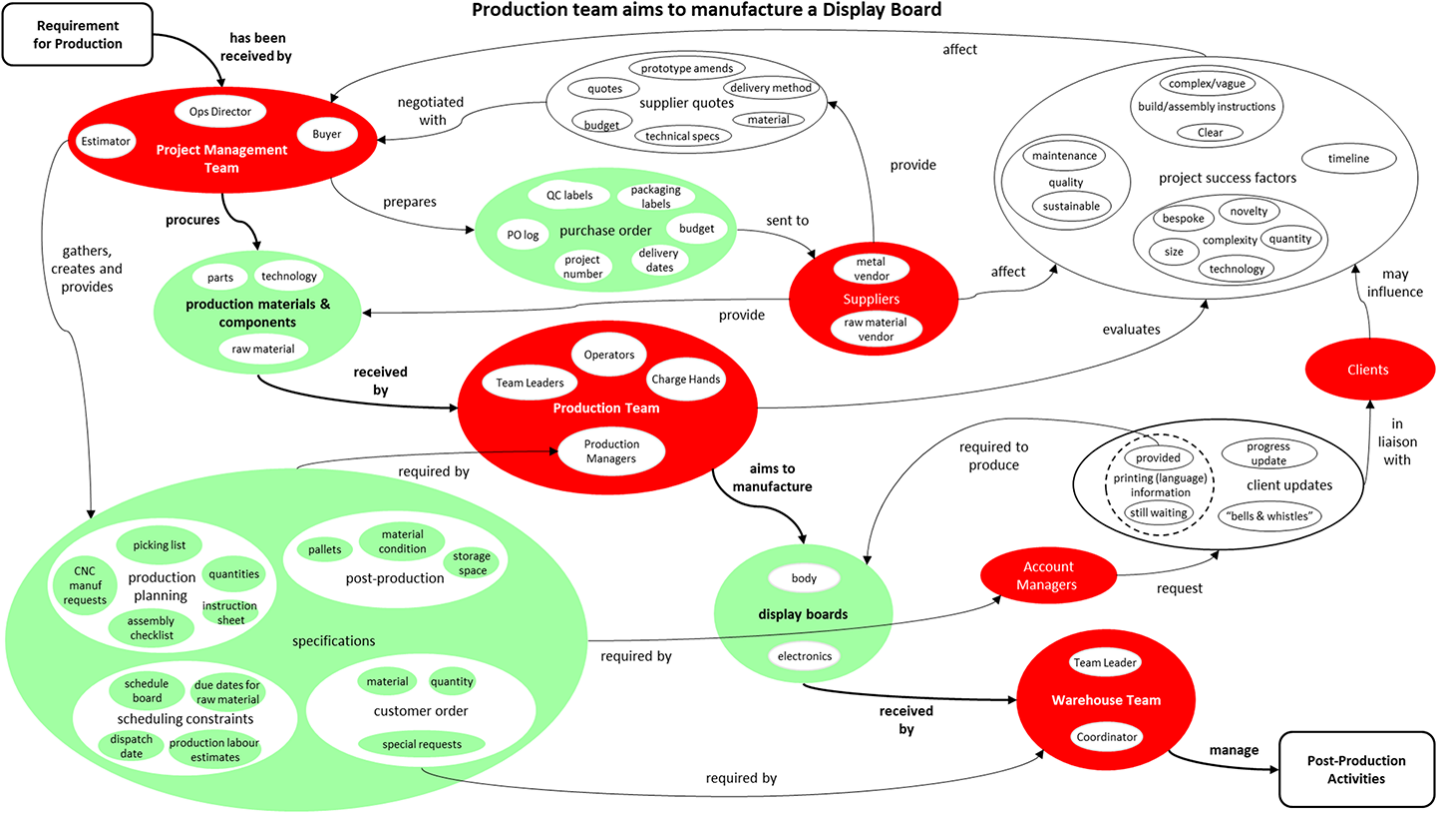
PrOH Model Depicting the Production Team aiming to Manufacture a Display Board

Having understood and defined the issues, it is imperative to plan for taking action towards improvement. Based on the process maps initially developed and the insights gathered, a core process statement (objective) has been agreed to build the PrOH Model as ‘Production team aims to manufacture a Display Board’ focusing on a typical client project. This statement takes a process perspective of production activities involved and captures the essence of what each stakeholder is individually aiming to achieve within each project. According to Clegg (2007) “it is imperative that each individual [PrOH] model should be given an objective to provide focus to a business process model. This objective should be reflected in its name which denotes the purpose why the process exists. This gives the exercise more validity and makes the application of systems thinking easier”. The action planning process ensured that all stakeholders had a shared understanding of the goals and their respective roles in achieving them. The PrOH Model, developed during this phase, captured the production process and reflected the objective of improving manufacturing efficiency, as depicted in Fig. 4. This PrOH Model instigated action, ensuring that the interventions were implemented and aligned with both the process requirements and the strategic goals of the production team.

#### Storyboarding

**Fig. 5:**
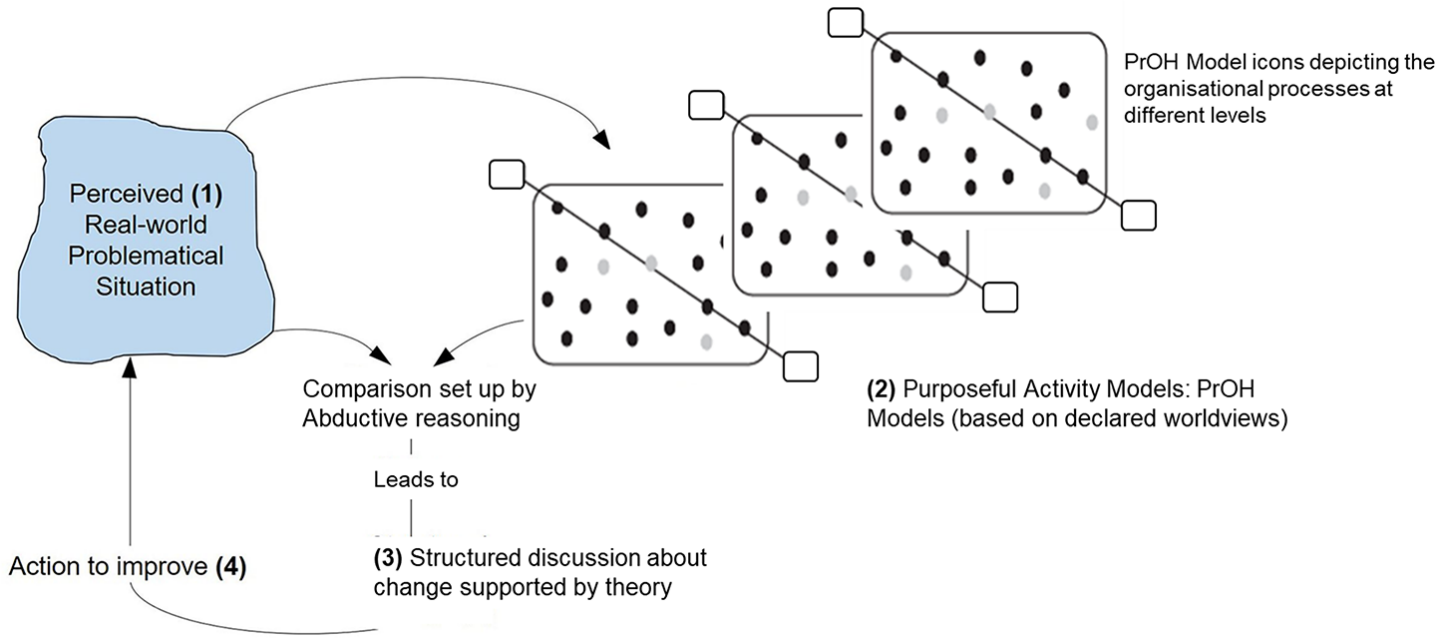
SSM’s Learning Cycle. (Adapted from Checkland and Poulter 2006)

During the PrOH Model storyboarding session, key participants including the Managing Director were invited to participate in a working session aimed at discussing current issues and suggesting potential improvements. The intention of this exercise from an SSM point of view is to *compare the purposeful activity models of change with the real world which sets up a structured discussion about change* as shown in Fig. 5. Storyboarding involves scripting a PrOH Model into incremental scenes and posing questions and or SSFs on each scene to tease out responses from participants in the workshop, helping to surface insights and stimulate dialogue on potential changes.

In a PrOH Model storyboard, questions were added onto every scene covering activity, dependencies and SSFs as suggested by Checkland (2006, pp. 49 & 77), such as: How is it done?, Who does it?, Could someone else do it?, How else could it be done?, Does it need to be done?, When and where is it done?, How do we judge it?

In every scene, participants are posed with questions which allowed them to participate in the discussion and highlight any issues as well as provide suggestions for improvement. Participants were allowed to input their ideas for improvements using post-it notes which were later analysed to be grouped into different clusters based on which the actions were designed.

### Action Taking

After PrOH Modelling storyboarding sessions, the Production department’s *desirable and feasible* process changes were identified, agreed and implemented as shown in Table 3.

**Table 3:** Examples of the SSFs for change for the production department over a period of 8 weeks

Systemic Success Factors of Change for the Production Department	Changes Achieved Through the Action Research Project
Inaccurate estimation of manufacturing and assembly times **(a. Scheduling)**	Accurate production times, increased predictability of workflow times. Achieved by implementing a system for recording prototype and manufacturing build times and defining manufacturing requirements. This allowed for more accurate estimation of production times and standardised procedures, leading to a more predictable workflow.
Lack of transparency between management, clients, and production personnel **(b. Communication)**	Standardised procedure in place, reduced order fulfilment time (on-time delivery) and repeat purchase rate. Achieved through the development of project forms for production stages, procedure book development, and reviewing the chain of command in the company structure. These changes clarified job roles, reduced waste, and improved communication, resulting in reduced order fulfilment time and increased customer satisfaction.
Absence of standardised quality control procedures **(c. Quality control)**	Clarity of job roles, reduced waste rate. Achieved by developing project forms for production stages and procedure book development, which provided clear guidelines and standardized procedures for quality control. This reduced waste and errors in production processes.
Poor communication channels leading to delays and errors **(b. Communication)** **(c. Quality control)**	Reduced quality issues and standardised production activities, reduced communication bottlenecks, reduced production errors and defect rate. Achieved through reviewing the chain of command in the company structure, adding new sections for receiving feedback in amendment forms.
Limited foresight into upcoming jobs for effective planning **(a. Scheduling)** **(e. Record Keeping and Logging)**	Production schedule attainment and increased visibility, increased availability of information (for benchmarking of future projects). Achieved by implementing Smartboard for planning.

These SSFs emerged from the iterative PrOH Modelling process, where key stakeholders collaborated to improve workflows, identify bottlenecks, and propose solutions. With PrOH Modelling workshops, insights gained from stakeholder discussions were translated into actionable changes. These SSFs reflect the combined perspectives and knowledge of the production team, which were used to design more efficient processes, ensuring that proposed changes were both realistic and aligned with organisational goals. Table 3 illustrates specific SSFs, highlighting the transition from modelling to practical implementation.

## Discussion: Situated Emancipatory Action Research - A New Framework

### Extending the SRA Model into a Multi-Cycle Situated Emancipatory Action Research Framework

**Fig. 6:**
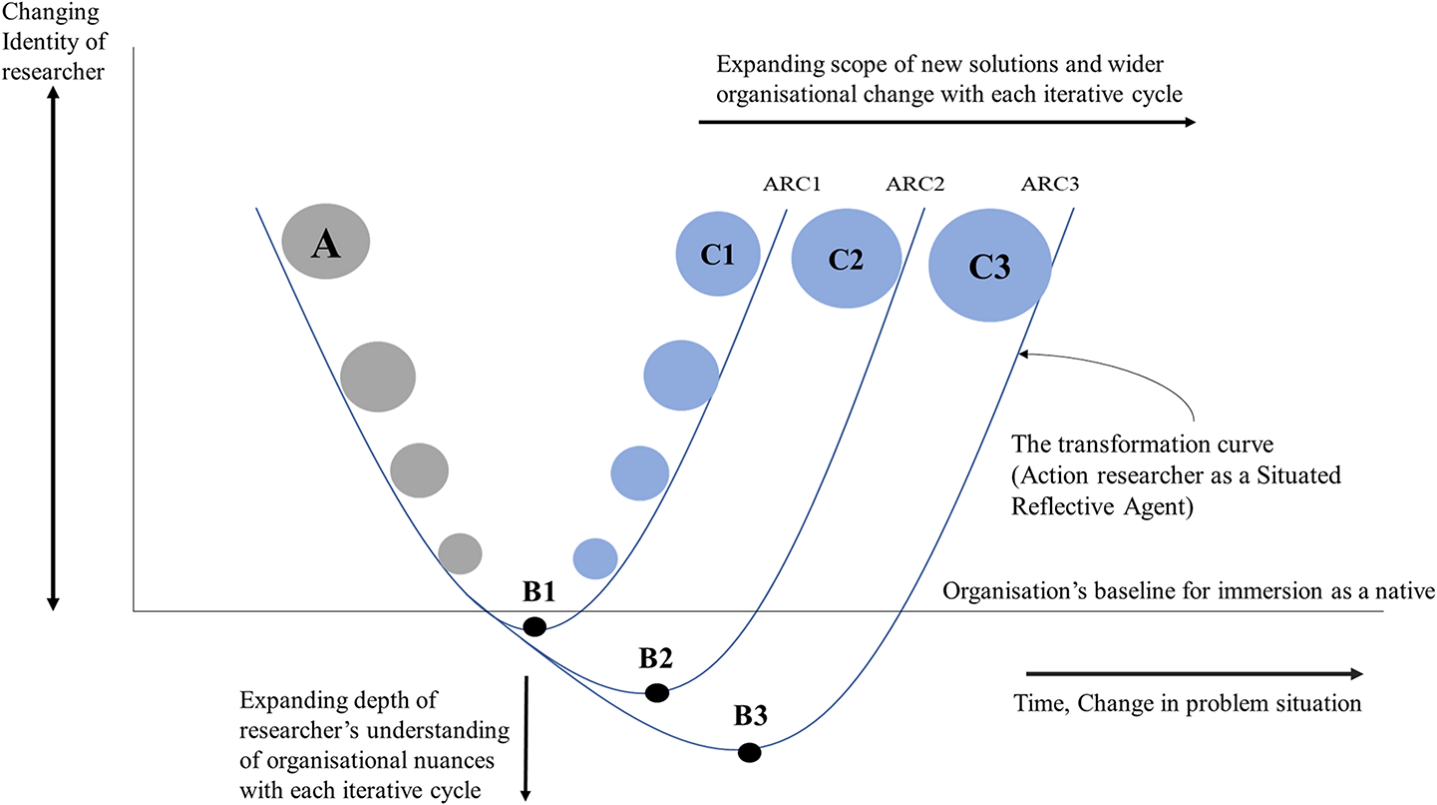
The Situated Emancipatory Action Research (SEAR) Framework

The Situated Emancipatory Action Research (SEAR) framework presented in Fig. 6 picks up where the SRA model leaves off, offering a logical next step for enacting successive, iterative cycles of inquiry in applied settings. Specifically, the SEAR framework integrates emancipatory intent, expanding cycles of PrOH Modelling, and flexible adaptation based on real-time learning. The emancipatory focus allows the SEAR framework to challenge restrictive organisational assumptions and structures through a process of critical empowerment, supported by PrOH Modelling. Expanding cycles enable progressively deeper understanding of the problem situation and broader impact over time. Integrating participatory methods like PrOH Modelling bring forth stakeholder perspectives through participation, knowledge co-creation and solution design.

### Enacting the SEAR Framework Through PrOH Modelling Methodology

This SEAR framework puts forth an iterative approach to action research that leverages off the PrOH Modelling Methodology (Clegg 2007; Clegg and Shaw 2008; Balthu and Clegg 2021). The SEAR framework facilitates continuous expansion of researchers’ understanding of organisational contexts and processes. The researcher immerses themselves in the organisational environment throughout each cycle, gaining deeper insights and fostering ongoing reflection. Specifically, in the first action research cycle (ARC 1), a researcher is immersed within the organisation through engaging employees in collaborative PrOH Modelling workshops. This immersion grants deep experiential insight into the initial problem situation allowing the researcher to recognise and confront their biases, as they interact with stakeholders and observe diverse perspectives. As a researcher then initiates ARC 2 and ARC 3 flowing directly from ARC 1, they can build upon the immersion (embedding) established through the first cycle’s PrOH activities, leading to subsequent design of new PrOH Models, action teams and solutions thereof.

In the diagram, the SEAR framework illustrates a reduced need for complete re-embedding of the researcher in each cycle, allowing more in-depth analyses at an accelerated pace (flowing down the Y-axis from point A) which gives stronger *agency* to the researcher through more insightful and impactful interventions. This expanding depth is depicted on the Y-axis of the model from points B1 to B2 and B3. Immersion in the problem situation grows along the Y axis, demonstrating the maturing of *situatedness*. With each iterative cycle, the researcher *deepens* their understanding of organisational nuances and tests new solutions toward addressing the defined problems.

Additionally, the X-axis width corresponds to the widening problem scope enabled as the cycles progress. Instead of retaining a static focus, the compounding insights gained from repeated PrOH Modelling iterations and SSF implementations encourage broader change ambitions across iterative cycles, which is depicted in the increasing size of C from C1 to C2 and C3. The point C indicates how the action researcher turns into a more effective *change agent* with greater *agency* to deliver highly relevant yet rigorous research-based change leading to higher levels of emancipation for client stakeholders due to their continual involvement throughout each ARC. More extensive organisational transformation becomes possible as researchers’ perspectives evolve. Points A, B and C as discussed in the SRA model (Fig. 2) remain central to the SEAR framework (Fig. 6) establishing the temporal nature of change in an action researcher’s *weltanschauung*, towards a more evolved and powerful agency of change embedded into the problem situation - yet cleansed of cognitivist biases and generating rigorous knowledge through cycles of action and reflection.

### The Phenomenon of Deepening and Widening in Successive ARCs

In ARC1, these researchers initiated their exploration within the Design department, closely collaborating with the design team. These researchers delved into the department’s activities and wider organisational routines, seeking to understand how each new project is received and delivered. However, the absence of a formal knowledge base within the company and the lack of clear documentation present significant barriers for developing a comprehensive picture of the departmental operations. These action researchers employed various research techniques such as stakeholder interviews, process mapping, and PrOH Modelling workshops to gain insights into the design process. All interviews, workshops, and meetings are audio recorded with the consent of participants, ensuring accurate capture of verbal exchanges. Additionally, written notes are taken during these sessions to capture key insights and non-verbal cues that may not be present in the audio recordings. Despite efforts to immerse in the design these researchers encountered limitations in fully grasping the intricacies of organisational norms, routines and processes due to lack of formal communication channels within the department. For example, the challenge of repeat instructions resulting in loss of productivity stems from lack of a simple version control system, and lack of structured client engagement. A designer noted “*Because what often happens is somebody will come back a week later and say I want you to change this drawing to this material or want you to change this…. whereas if we could identify that before they’ve been issued*,* it would avoid having to make revisions and issuing new revisions”.* ARC1 highlights the importance of developing a comprehensive understanding of the organisation’s dynamics, despite initial constraints, to establish a foundation for subsequent cycles of inquiry.

ARC2 marks a pivotal advancement as the research focus shifts to the production department, enabling a deeper understanding (point B2) of underlying systemic success factors. Within the production environment, characterized by a focus on efficiency and process optimisation, the researcher delved into operational intricacies and supply chain dynamics. Through extensive engagement with stakeholders and iterative modelling sessions, hidden complexities that shape organisational behaviour were tapped into gaining insights not previously accessible. For example, a discussion around some poor norms and routines was highlighted, in the words of a Project Manager, as, *“Unfortunately [researcher name]*,* we call it the ‘Design Co. way’ [pseudonym]*,* basically we get ‘first sign-off’ but sometimes things get missed at that point. Because the project managers haven’t been told*,* or maybe it’s been forgotten*,* and we rushed to sign-off and then we’ve got 50 to 100 to 1000 boxes sitting there and we open them up and we do the retrospective changes. It’s the Design Co. way. It’s a bit of a standing joke*,* but it’s quite an issue”*. Such access to deeper organisational practices and cultural nuances led to deepening of the researcher’s understanding of the problem situation, reflected in the SEAR framework and an increasing *depth* of knowledge at point B2.

Concurrently, ARC2 also sees a *widening* scope of organisational change initiatives reflected through point C2. Building upon the foundational knowledge acquired in ARC1, researchers implemented solutions such as developing standardised procedures through the procedure book for recording prototype and manufacturing build times, establishing clear job roles through project forms, and enhancing communication channels through new feedback mechanism. These changes resulted in tangible improvements, including more accurate production time estimation, reduced order fulfilment time, better quality control, improved communication flow, and increased visibility into upcoming jobs. This expanded scope of changes from the previous ARC1 reflects a broader and more holistic approach to organisational change, driven by the deeper understanding attained through ARC2’s rigorous exploration of production processes and dynamics.

ARC3 presents further advancement as the research focus extends to the Shipping department, deepening the researcher’s comprehension (point B3) of the problem situation. The Shipping department’s pivotal role in logistics and distribution offers valuable insights into broader systemic challenges related to inventory management, transportation, and customer service. Collaborative workshops and action planning sessions with shipping personnel facilitated knowledge exchange and co-creation of solutions aimed at enhancing operational efficiency and customer satisfaction. This phase allowed the researcher to gain a deeper understanding of the complexities involved in managing shipping operations, managing inventory levels, and ensuring timely deliveries, which are all intrinsically linked to the issues identified in ARC1 and ARC2. For example, some of the complexities involved in shipping are highlighted by a member of staff as “*We’ve got a list basically of all the different elements that we need to be able to ship abroad. And we just keep sending it to the account managers and say that these are the vital information*,* these are the*,* you know the key parts that we need*,* and they’ll just go back to the customer. But sometimes it could take a week*,* sometimes shipments get held up because of it*,* sometimes they end up with our hauliers or customs. They’ll say we can’t clear it; we haven’t got the right information. Then they’ll have to put it in there for a couple of days while we get the information. And then obviously the process starts again*,* and they get through customs and all the rest of it. But yeah*,* it’s there are issues*,* a lot of issues with it”.* This issue directly links to broader issue of information management and poor communication channels previously identified in Design and Production departments.

ARC3 sees a widening scope of organisational change initiatives (point C3). Building upon the foundational knowledge acquired in previous cycles, researcher implemented strategic initiatives such as technology adoption and operational redesign. This expanded scope encompasses efforts to streamline shipping processes and leverage technology to enhance customer service capabilities. For example, the newly piloted customer information portal and the Customer Relationship Management (CRM) system have potential to eliminate the challenges discussed above. By integrating emancipatory intent, situating the researcher in context, and expanding cycles of inquiry, the SEAR framework aims to address common criticisms of action research. It supports rigorous, empowering interventions that foster emancipation of both the *researcher* and the *researched* delivering authentic transformation. Emancipation for the *researcher* occurs through the process of *deepening* along the Y-axis in successive ARC iterations from B1 to B3, allowing them to gain deeper insights into organisational dynamics. Emancipation for the *researched* occurs through the process of *widening* along the X-axis as a result of staff participation in collaborative problem-solving activities and the exploration of alternative solutions leading to organisational changes.

The above two facets of the SEAR framework, *deepening* and *widening*, align with calls for action research to solve real-world problems, integrate diverse views, build change capacity, and catalyse researcher agency and participant evolution through critical reflection (Bradbury-Huang 2010). The SEAR framework therefore provides methodological details to translate the SRA model into an extended and more comprehensive framework for conducting emancipatory action research.

### Core Principles of SEAR Framework: Addressing Limitations and Achieving Emancipatory Action Research

This SEAR framework aligns with foundational thinking on action research and its core principles put forth by previous research (Eden and Huxham 1996; Reason & Bradbury 2001; Bradbury-Huang 2010). Specifically, Bradbury-Huang advocates core principles of participation, solving real-world problems, integrating diverse views, and building capacities for change. In line with Mode 3 action research, as discussed by Grundy (1988), the SEAR framework emphasises the active participation of both the researcher and the researched, fostering a collaborative, reflexive process that not only aims for problem-solving but also promotes emancipation and transformation for both parties involved. The PrOH Modelling Methodology at the heart of the SEAR framework fulfils these criteria of participation, solving problems, plurality, and change. Furthermore, the researcher’s sustained embedded presence across action research cycles facilitates their own cognitive emancipation as a situated reflective agent. The phrase ‘*cognitive emancipation’* is coined here to refer to the process by which individuals free themselves from precepts, cognitive biases, and constraints induced by the *multiple interacting perceptions of reality*, allowing for more open-mindedness, critical thinking, and reflexivity, which have been discussed above as some of the essential ingredients for undertaking sound action research. Measuring cognitive emancipation is inherently challenging, as it involves subjective changes in mindset, biases, and reflexivity. However, these changes can be assessed through the volume and quality of SSFs generated from stakeholder interactions during ARCs. The extent to which these SSFs were successfully implemented and their subsequent impact on organisational performance can be measured.

The SEAR framework acts as a guide for action researchers. Below are the core principles of the SEAR framework, each addressing a critical limitation of CAR while enabling two-fold emancipation for both the researcher and the researched.

***a. Transforming Researcher Identity: Overcoming Detached Observation***

Traditional CAR positions the researcher as a detached observer, limiting engagement with the organizational context and creating a gap between theory and practice (Kemmis 2008). Such external positioning can hinder the researcher’s ability to grasp the complexities of organizational dynamics, leading to a static and restricted researcher identity.

SEAR addresses this by fostering the evolution of the researcher’s identity. Over successive ARCs, a researcher transitions from an outsider to an insider, deeply involved in the problem-solving process. Through PrOH Modelling workshops and stakeholder interactions, the researcher’s identity becomes more fluid blending in the organisation with people and processes, getting to grips with the systems, understanding the culture allowing for *cognitive emancipation* (Bradbury and Reason 2001). This shift empowers researchers to engage more authentically, making them more active agencies of change rather than passive observers.

***b. Situating the Researcher: Embedding Within the Organizational Context***

Traditional CAR positions a researcher outside the organizational context, limiting their understanding of deeper issues at play within the organisation. This external perspective restricts the researcher’s ability to engage intimately with organizational culture and processes, which are critical for generating meaningful insights and solutions (Haraway 1988). The SEAR framework overcomes this limitation by ensuring that the researcher is deeply embedded within the organizational environment, fostering a more intimate understanding of its practices, challenges, and interactions. Over successive ARCs, a researcher becomes progressively integrated into an organisational culture. For instance, in ARC2 in the design company, the researcher’s continuous engagement with the production department and elicitation of issues allowed them to identify operational bottlenecks and workflow inefficiencies. Through PrOH Modelling workshops and stakeholder interviews, the researcher immersed themselves in the organisation’s systems, observing the challenges firsthand and gathering rich, context-specific data. This level of immersion helped the researcher transcend a superficial understanding and develop situated knowledge that is specific to the organisation. As researchers’ situatedness deepens, they gain the capacity to offer more relevant, actionable solutions, ensuring that interventions are both practical and tailored to the specific needs of an organisation. To determine when immersion is “deep enough,” the researcher relies on data and theoretical saturation, when no new insights or patterns emerge (Glaser and Strauss 1967). Action research accepts that social phenomena evolve over time, and ending research is ultimately a judgment call (Checkland and Holwell 1998). The cycle concludes when the researcher deems significant learning has occurred, with further exploration no longer yielding new knowledge, ensuring interventions are tailored to the organisation’s needs and the outcomes are context specific.

***c. Engaging in Reflexivity: Reflecting-in-Action***

Traditional CAR often relegates reflection to a post-action chore, where the researcher reflects on their findings and actions after-the-fact. This temporal separation reduces the immediacy and effectiveness of the reflective process, hindering a researcher’s ability to make real-time adjustments to interventions (Schön 1983). The SEAR framework addresses this limitation by embedding reflection-in-action throughout each research cycle. In each ARC, a researcher continuously reflects on their role and interventions in the moment, making real-time adjustments based on emerging insights. For example, in ARC3, as these researchers engaged with the Shipping department, they encountered unforeseen communication breakdowns that delayed shipments. Rather than waiting until the next cycle to address the issue, these researchers immediately reflected on the problem during a PrOH Modelling session with the staff, prompting a real-time solution involving enhanced feedback loops between the shipping team and account managers. By incorporating real-time reflection, these researchers ensured that interventions remained relevant and responsive to the changing dynamics of the organisation.

Table 4 below provides an account of an action researcher’s reflection on their journey through successive ARCs. The SEAR framework provides methodological guidance and principles rather than a rigid process to support flexible, responsive adaptation to organisational complexities. This framework leverages on participatory and intervention-based approach imbued with PrOH Modelling to facilitate engagement of stakeholders and increased agency of an action researcher in co-creation of solutions by tapping into their collective experience and knowledge.

While SRA users will understand the need for situatedness and reflection in action, the SEAR framework provides extended scaffolding to fully enact multiple ARCs of organisational inquiry. This empowers action researchers to confront endemic challenges like reactivity, bias, and lack of participant-voice through embedded and emancipatory practice. In essence, the SEAR framework signifies a logical evolution of the SRA model toward more rigor, agency with real-world impact.

**Table 4:** Evolution of researchers’ identity and situatedness across action research cycles

Action Research Cycle (ARC)	Deepening of Researchers' Understanding (B)	Widening of Organisational Change (C)	Researchers’ Identity & Situatedness	Reflection on Emancipation
**ARC1**	**Initial Understanding**: Through PrOH workshops and stakeholder interviews, the researchers gain initial insights into design inefficiencies. However, surface-level understanding remains due to limited organisational documentation.	**Narrow Focus**: Focused on design process inefficiencies, such as delays in project sign-offs and lack of version control. The researchers propose a more structured process for documentation.	**Outsider Role**: Initially, the researchers observe the design team, focusing on process inefficiencies without fully understanding the team’s internal dynamics.	**Reflection**: The researchers acknowledge limitations in their understanding due to lack of formal communication channels. Recognise the need for deeper engagement and begin to question their position as outsiders.
**ARC2**	**Deeper Immersion**: PrOH Modelling sessions with the production team reveal deeper organisational issues such as missed communications during the design process. The researchers begin to understand how production bottlenecks are interconnected with earlier stages.	**Expanding Focus**: The researchers expand their scope to include cross-departmental issues, working on standardising procedures, developing project forms, and implementing a feedback loop to improve communication.	**Transition to Insider**: As the researchers become more involved in team meetings and workshops, they move from an outsider to an insider, building trust and facilitating change.	**Reflection**: The researchers reflect on their growing involvement and how their increasing role impacts the project’s direction. Researchers' understanding deepens as they actively contribute to the creation of standardised procedures.
**ARC3**	**Comprehensive Understanding**: The researchers facilitate workshops in the shipping department, utilising PrOH Modelling to explore systemic success factors (SSFs) such as information delays in shipments. The insights gained help connect issues across departments (Design, Production, and Shipping).	**Broader Organisational Change**: Based on prior insights, the researchers propose system-wide changes such as the adoption of a CRM system and a customer information portal to enhance communication and operational efficiency.	**Fully Integrated Change Agent**: The researchers are now fully embedded within the team, facilitating the collaboration between all departments. Researchers play a pivotal role in co-creating solutions and are seen as a key partner.	**Reflection**: The researchers reflect on their transition from an external observer to active agents of change. Researchers recognise how their immersion in organisational culture (going native) has allowed them to address deeper systemic issues.
**Post-ARC**	**Strategic Understanding**: In the final stage, PrOH Models from departmental workshops are consolidated into a higher-level strategic model. The researchers collaborate with senior managers, including the managing director, to address systemic issues that affect the entire organisation (e.g. staffing issues, reinvestment, capacity building).	**Systemic Transformation**: The strategic PrOH Model is used to guide long-term changes, including the restructuring of workflows and technology adoption, leading to improved efficiency and customer service across all departments.	**Trusted Partner and Co-Creator**: The researchers are now considered an integral part of the organisational team, contributing to long-term strategic planning and organisational development.	**Reflection**: The researchers reflect on their full immersion into the organisation’s systems and processes, noting the transformation in their role from researchers to co-creators. Researchers feel empowered through the collaborative approach and acknowledge the broader organisational impact of their work.

### Limitations of the SEAR Framework

The SEAR framework does not position itself as a definitive solution, but rather as a framework for nurturing reflective action research. It serves as a map to track and understand a researcher’s cognitive journey, which is central to the iterative cycles of action and reflection within each ARC of the SEAR framework. By focusing on a researchers’ evolving identity and deepening engagement with the organisational context, SEAR encourages continuous immersion and reflection-in-action. However, there are limitations to the SEAR framework, which are as follows:

- The framework assumes a researcher is able to stay embedded within an organisation across multiple action research cycles. But maintaining access and engagement over an extended period of time may be challenging in some applied settings.
- There is an assumption that each action research cycle (ARC) will build neatly upon the last, but organisational realities are often messy. Findings and plans from one cycle may lose relevance as contexts shift.
- While the framework aims to expand scope over successive cycles, there is a risk of trying to expand too quickly without properly consolidating lessons and capabilities from prior cycles.
- The X and Y axes are theoretical concepts that may be difficult to quantify in practice across different projects. This could limit the ability to clearly track progression along these axes.
- The framework is conceptual and developed as a methodological guidance similar to Davison et al. (2004) for action researchers. Individual researchers should exercise discretion in adapting it to varied organisational settings and turning it into actionable research design.
- Emancipation in action research is inherently qualitative and subjective, making it difficult to measure with precision. Capturing shifts in participants’ perspectives, their development of agency, and their ownership of solutions requires continuous dialogue and reflection, which are challenging to quantify. While the SEAR framework emphasizes emancipation, further research is needed to develop effective methods for evaluating its impact on participants.

The SEAR framework presents a logical approach to iterative action research, but may need additional development regarding real-world application, flexibility, pacing, integrating evaluation, metrics, and translation into concrete methods to maximise its utility. However, the structure is guided by the core principles of quality in action research as highlighted by various suggestions from authors about ensuring quality in action research. For example: three criteria of rigour, reflection and relevance by Pasmore et al. (2007); four quality dimensions of organisation development through action research (Coghlan and Shani 2014); five quality criteria proposed by Heikkinen et al. (2007); and 15 characteristics of good action research (Eden and Huxham 2006).

## Conclusion

The Situated Reflective Agent (SRA) model emerged from an action research project conducted in a legal services firm. The principles of the SRA model were then tested and evolved through engagement with a manufacturing firm. The manufacturing case demonstrated how successive cycles of participatory modelling could *deepen* understanding and *widen* the scope of intervention. This inspired formalising the Situated Emancipatory Action Research (SEAR) framework to translate the SRA ethos into a comprehensive iterative approach. This framework is a result of years of application of the novel version of SSM called the PrOH Modelling Methodology (Clegg 2007); which has been rigorously used in different organisational settings including the case studies presented here, for designing and delivering organisational change rooted in the principles of systems thinking. Each application has advanced the SEAR framework based on the distinctive organisational context, for example the legal services and, the manufacturing operations. With each iteration, the rigor and utility of the framework improved by confronting the dynamics, constraints, and diversity posed by different organisations.

The SEAR framework offers a synthesis of diverse theoretical perspectives in reflective practice and action research. By drawing on Schön’s (1983) *reflection-in-action*, collaborative inquiry principles, and systems thinking through PrOH Modelling, the SEAR framework provides a robust response to the identified limitations of CAR. It captures the cognitive emancipatory journey of an action researcher, embracing not only *detached observation* of Schein (1999) but also gaining *immersive engagement* and developing *tacit knowledge* of Polanyi (1966) thus enhancing the depth and authenticity of *reflection-in-action*. By doing so, the framework aligns with Bradbury and Reason’s call for a richer, more contextually embedded exploration of ways of *thinking* and *knowing* within the action research process. Therefore, this paper not only contributes to the refinement of the Canonical Action Research methodology but also aligns with broader discussions on the evolving nature of reflective practice, and researcher’s identity in complex and dynamic organisational contexts (Yanow and Tsoukas 2009; Hadjimicheal et al. 2024).

### Recommendations for Future Research

The recommendations for future research aim to refine and enhance the Situated Emancipatory Action Research (SEAR) framework in the context of participatory action research methodologies. Conducting additional case studies across diverse organisational contexts is proposed to further validate and generalise the utility of the framework. Different settings, including the public sector, non-profit organisations, community-based entities, and virtual organisations, could provide insights into necessary adaptations. Experimentation with alternative participatory modelling approaches beyond the PrOH Modelling Methodology is suggested. This involves identifying suitable modelling methodologies for different contexts and subsequently comparing their efficacies. Devising metrics to assess the expansion of scope, an increase in the depth of understanding, and the evolution of researcher identity across cycles, as defined in the framework, will need to be considered.

## Data Availability

No datasets were generated or analysed during the current study.

## References

[CR1] Ackoff RL (2006) Why few organizations adopt systems thinking. Syst Res Behav Sci 23:705–708

[CR2] Alvesson M, Karreman D (2004) Interfaces of control: technocratic and socio-ideological control in a global management consultancy firm. Acc Organ Soc 29(3):423–444

[CR3] Alvesson M, Sköldberg K (2009) Reflexive methodology: new vistas for qualitative research. SAGE, London

[CR4] Argyris C, Schon D (1974) Theory in practice: increasing professional effectiveness. Jossey Bass, San Francisco

[CR5] Balthu K, Clegg B (2021) Improving professional service operations: action research in a law firm. Int J Oper Prod Manage 41(6):805–829

[CR6] Blau PM, Scott WR (1962) *Formal Organizations: A Comparative Approach*, Chandler

[CR7] Bradbury H (2015) The SAGE handbook of action research. SAGE Publications Ltd

[CR8] Bradbury H, Reason P (2001) Conclusion: broadening the bandwidth of validity: issues and Choice-points for improving the quality of action research. In: Reason P, Bradbury H (eds) The handbook of action research. SAGE, London/Thousand Oaks, CA, pp 447–456

[CR9] Bradbury-Huang H (2010) What is good action research? Why the resurgent interest? Action Res 8(1):93–109

[CR10] Carr W, Kemmis S (1986) Becoming critical: education, knowledge & action research. Deakin University, Victoria

[CR11] Castelló M, McAlpine L, Sala-Bubaré A, Inouye K, Skakni I (2020) What perspectives underlie ‘researcher identity’? A review of two decades of empirical studies. High Educ 81(4):567–590

[CR13] Checkland PB (1981) Systems thinking, systems practice. Wiley, Chichester

[CR14] Checkland PB (1985) From optimizing to learning: A development of systems thinking for the 1990s. J Oper Res Soc 36(9):757–767

[CR12] Checkland P (1991) From framework through experience to learning: the essential nature of action research. In: Nissen H-E, Klein HK, Hirschheim R (eds) Information systems research. Elsevier, Amsterdam

[CR18] Checkland PB (1995) Soft systems methodology and its relevance to the development of information systems. In: Stowell FA (ed) Information systems provision: the contribution of soft systems methodology. McGraw-Hill, Maidenhead, UK

[CR69] Checkland P, (1999) Systems thinking, systems practice: a 30-year retrospective. Chichester, New York: John Wiley.

[CR15] Checkland P, Holwell S (1998) Action research: its nature and validity. Systemic Pract Action Res 11:9–21

[CR16] Checkland P, Poulter J (2006) Learning for action: A short definitive account of soft systems methodology and its use, for practitioners, teachers and students. John Wiley & Sons Ltd, Chichester

[CR17] Checkland P, Scholes J (1990) Soft systems methodology in action. Wiley

[CR19] Churchman CW (1979) *‘The Systems Approach’*. Dell New York

[CR20] Clegg BT (2007) Building a holarchy using business process orientated holonic (PrOH) modeling. IEEE Syst Man Cybernetics: Part A 31(1):23–40

[CR22] Clegg B (2020) Improving systemic success factors in a University: using the proh modelling methodology. Bus Process Manage J 26(2):630–654

[CR23] Clegg B, Shaw D (2008) Using process-oriented holonic (PrOH) modelling to increase Understanding of information systems. Inform Syst J 18(5):447–477

[CR21] Clegg BT, Balthu KC, Morris G (2020) Changing professional service archetypes in a law firm using process orientated holonic (PrOH) modelling. Knowl Manage Res Pract 18 1:pp38–52

[CR24] Coghlan D, Brannick T (2014) *Doing Action Research in Your Own Organization*, 4th edition, SAGE

[CR25] Coghlan D, Shani A (2014) Creating action research quality in organization development: rigorous, reflective and relevant. Systemic Pract Action Res 27(6):523–536

[CR26] Coughlan P, Coghlan D (2002) Action research for operations management. Int J Oper Prod Manage 22(2):220–240

[CR68] Creswell JW, Creswell JD (2017) Research design: Qualitative, quantitative, and mixed methods approaches. 4th ed. Newbury Park: Sage.

[CR27] Cunliffe AL (2003) Reflexive inquiry in organizational research: questions and possibilities. Hum Relat 56:981–1001

[CR28] Davison RM, Martinsons MG, Kock N (2004) Principles of canonical action research. Inform Syst J 14(1):65–86

[CR29] Davison RM, Martinsons MG, Ou CXJ (2012) The roles of theory in canonical action research. MIS Q 36(3):763–786

[CR30] Denzin NK, Lincoln YS (eds) (2005) The Sage handbook of qualitative research, 3rd edn. SAGE, Thousand Oaks, CA

[CR31] Eden C, Huxham C (1996) Action research for management research. Br J Manag 7(1):75–86

[CR32] Eden C, Huxham C (2006) Researching organizations using action research. In: Clegg SR, Hardy C, Lawrence T, Nord WR (eds) The SAGE handbook of organization studies, 2nd edn. SAGE, London, pp 607–629

[CR33] Eikeland O (2012) Action research? - Applied research, intervention research, collaborative research, practitioner research, or praxis research?? Int J Action Res 8(1):9–44

[CR34] Glaser B, Strauss A (1967) The discovery of grounded theory: strategies for qualitative research. Sociology, Mill Valley, CA

[CR35] Greenwood DJ, Levin M (1998) Introduction to action research: social research for social change. Sage Publications, Inc.

[CR36] Griffin D, Shaw P, Stacey R (1999) Knowing and acting in conditions of uncertainty: A complexity perspective. Systemic Pract Action Res 12(3):295–309

[CR37] Grundy S (1988) Three Modes Of Action Research, in Kemmis,S. and McTaggert,R. (eds) (1988). The Action Research Reader (3ed). Geelong: Deakin University Press

[CR38] Hadjimicheal D, Ribeiro R, Tsoukas H (2024) How does embodiment enable the acquisition oftacit knowledge in organizations? From Polanyi to Merleau-Ponty. Organ Stud 45:545–570

[CR39] Hakkarainen V, Ovaska U, Soini K, Vainio A (2023) ‘Being’ and ‘doing’: interconnections between researcher identity and conceptualizations of sustainability research. Sustain Sci 18(7):2341–2355

[CR40] Haraway D (1988) Situated knowledges: the science question in feminism and the privilege of partial perspective. Feminist Stud 14(3):575–599

[CR41] Heikkinen HLT, Huttunen R, Syrjala L (2007) Action research as narrative: five principles for validation. Educational Action Res 15(1):5–19

[CR42] Heron J, Reason P (2008) Extending epistemology within a Co-Operative inquiry. In: Reason P, Bradbury H (eds) Handbook of action research: participative inquiry and practice. SAGE, London, pp 366–370

[CR43] Kemmis S (2008) Critical theory and participatory action research. In: Reason P, Bradbury H (eds) Handbook of action research: participative inquiry and practice. SAGE, London, pp 366–370

[CR70] Koestler A (1967) The ghost in the machine. London: Penguin Books. (Original work published 1967).

[CR44] Lorsch JW, Mathias PF (1987) When professionals have to manage. Harvard Business Rev 65(4):78–83

[CR45] Løwendahl B (1997) Strategic management of professional service firms. Copenhagen Business School, Copenhagen

[CR46] Marshall J (2004) Living Systemic Thinking: Exploring Quality in First-Person Action Research, Action Research, 2004 Vol. 2 No.1, pp. 305–325

[CR47] McCutcheon G, Jung B (1990) Alternative perspectives on action research. Theory into Pract 29:144–151

[CR48] McNiff J (2013) Action research: principles and practice. Taylor & Francis Group, London

[CR49] McTaggart R (1994) Participatory action research: issues in theory and practice. Educational Action Res 2(3):313–337

[CR50] Morris T, Malhotra N (2002) ‘Towards Managerialism: Analysing the Process of Change in Professional Service Organisations’, Paper presented at the 4th Biennial Workshop on Professional Service Firms, Alberta, August

[CR51] Nordenflycht AV (2010) What is a professional services firm?? Towards a theory and classification. Acad Manage Rev 35(1):155–174

[CR71] Pasmore W, Woodman R, Simmons A (2007) Toward a more rigorous, reflective, and relevant science of collaborative management research. In: Shani ABR, Mohrman SA, Pasmore WA, Stymne B, Adler N (Eds.), Handbook of collaborative management research (pp. 567–582). Sage Publications.

[CR53] Polanyi M (1966) The Tacit dimension. Routledge & Kegan Paul, London

[CR54] Rapoport RN (1970) Three dilemmas in action research. Hum Relat 23(6):499–513

[CR55] Reason P (1994) Three approaches to participatory inquiry. In: Denzin K, Lincoln S (eds) Handbook of qualitative research. SAGE, Thousand Oaks, CA, pp 324–339

[CR56] Reason P, Bradbury H (eds) (2001) Handbook of action research: participative inquiry and practice. SAGE, London

[CR57] Sampson SE (2012) Visualizing service operations. J Service Res 15(2):182–198

[CR58] Schein EH (1999) Process consultation revisited: Building the helping relationship. Addison-Wesley, Reading, MA

[CR59] Schön DA (1983) The reflective practitioner. Temple Smith, London, England

[CR60] Stowell FA (2024) The Art of soft systems inquiry: Retracing the footsteps of churchman and checkland. Systemic Pract Action Res Systemic Pract Action Res Vol 37:1123–1140

[CR61] Susman GL, Evered RD (1978) An assessment of the scientific merits of action research. Adm Sci Q 23(4):582–603

[CR62] Taylor S (2004) Presentational form in first person research: Off-line collaborative reflection using Art. Action Res 2(1):71–88

[CR63] Torbert WR (1998) Developing wisdom and courage in organizing and sciencing. In: Shrivastva S, Cooperrider DL (eds) Organizational wisdom and executive courage. New Lexington, San Francisco, CA, pp 222–253

[CR64] Weiner-Levy N, Abu-Rabia-Queder S (2012) Researching my people, researching the other: field experiences of two researchers along shifting positionalities. Qual Quant 46(4):1151–1166

[CR65] Wilson B (2001) *Soft systems methodology: conceptual model Building and its contribution*. Wiley, Chichester

[CR66] Yanow D, Tsoukas H (2009) What is Reflection-In-Action? A phenomenological account. J Manage Stud 46(8):1339

[CR67] Yin RK (2017) Case study research and applications, 6th edn. SAGE Publications US

